# Nationality, Gender, Age, and Body Mass Index Influences on Vitamin D Concentration among Elderly Patients and Young Iraqi and Jordanian in Jordan

**DOI:** 10.1155/2016/8920503

**Published:** 2016-03-24

**Authors:** Hanan Al-Horani, Wael Abu Dayyih, Eyad Mallah, Mohammed Hamad, Mohammad Mima, Riad Awad, Tawfiq Arafat

**Affiliations:** ^1^Faculty of Pharmacy and Medical Sciences, University of Petra, Amman 00962, Jordan; ^2^College of Sciences and Health Professions, King Saud Bin Abdulaziz University for Health Sciences, Jeddah, Saudi Arabia

## Abstract

Vitamin D is necessary for maintaining and regulating calcium levels; thus, insufficiency of vitamin D increases the risk of many chronic diseases. This study aimed to examine vitamin D levels among Jordanian and Iraqi volunteers and find the relation between vitamin D level and lipid profile patients. Vitamin D levels were evaluated using enzyme-linked immunosorbent assay. For young healthy group subjects, vitamin D levels were 20.60 ± 5.94 ng/mL for Jordanian and 27.59 ± 7.74 ng/mL for Iraqi. Vitamin D concentrations for young males and females were 25.82 ± 8.33 ng/mL and 21.95 ± 6.39 ng/mL, respectively. Females wearing hijab were 20.87 ± 6.45 ng/mL, while uncovered females were 23.55 ± 6.04 ng/mL. For >40 years Iraqi subjects, vitamin D level for healthy was 29.78 ± 9.49 ng/mL and 23.88 ± 7.93 ng/mL for hyperlipidemic subjects. Vitamin D levels for overweight and obese healthy groups were significantly higher (*P* < 0.050) than those for the hyperlipidemic patients groups. Vitamin D levels for males were significantly higher than females and were significantly higher for healthy than those hyperlipidemic Iraqi patients. These findings showed that vitamin D levels are affected by age, nationality, gender, and health statues and highlight the importance of vitamin D supplementation for groups with low levels particularly old, hijab wearing females, and hyperlipidemic groups.

## 1. Introduction

Vitamin D is a fat soluble hormone that plays essential role in calcium homeostasis and mineralization of bones [1]. Vitamin D is unique, in terms of its metabolism and physiologic features. Human dependence on both endogenous syntheses (activation through exposure to ultraviolet light) accounts for about 90% of vitamin D (vitamin D3) and exogenous sources (diet, primarily fortified foods) to meet biological requirements (vitamins D2 and D3) [2–4].

Vitamins D3 (cholecalciferol) and D2 (ergocalciferol) are metabolized in an identical manner in the liver to 25-hydroxyvitamin D [5], by the enzyme cytochrome P450 (vitamin D 25-hydroxylases) to 25-hydroxyvitamin D3, which is the most abundant form of vitamin D in the circulation. Further hydroxylation of 25-hydroxyvitamin D to 1, 25(OH)2D (active vitamin D) by the 1*α*-hydroxylase enzyme occurs in the kidney [6]. A circulation of approximately 10–15 days half-life of 25(OH)D [7] makes it the ideal measure for vitamin D, although the concentration of 25(OH)D in the serum was 8–60 ng/mL or 20–150 nmol/L [7].

Many studies reported that vitamin D low levels negatively affect bone mineralization causing rickets in children and osteomalacia in adults [4, 8]. In addition, vitamin D insufficiency is associated with other diseases; chronic kidney disease (CKD) gives rise to secondary hyperparathyroidism (SHPT) which can lead to loss of bone density and elevated rates of fracture in renal patients [9], common cancers [3], autoimmune disorders [10, 11], multiple sclerosis [12], cardiovascular disease [13], lung function, and asthma [14]. Also, epidemiological studies show that low blood levels of 25-hydroxyvitamin D (25(OH)D, a marker of vitamin D status), are linked with an increased risk of type 2 diabetes [14].

Besides, higher levels of 25(OH)D are associated with a healthier lipid profile [15, 16]. However, its levels were found to be low in subjects with hypertriglyceridemia and hypercholesterolemia [16]. These studies however may be unable to differentiate the causation from association because of the possible uncontrolled confusing and inverse causation.

The growing data from studies conducted on young adults, elderly persons, and youth in different countries showed that vitamin D deficiency is not recognized and is not a predominant health problem [17]. Vitamin D status varies among countries according to latitude, dietary intake of fish and liver oil, season, and skin pigmentation. Higher 25(OH)D levels in Northern Europe compared with Southern Europe have only been found in elderly people. In adolescents, 25(OH)D levels are higher in the south of Europe than in the northern parts of Europe [18, 19].

In Middle East countries, high variation in serum 25(OH)D levels was revealed. The lowest level of 3.6 ng/mL (9 nmol/mL) was seen in a study conducted on older persons in Saudi Arabia [20]. Mallah et al. reported a strong correlation between the levels of 25(OH)D and clothing in Jordanian women [21]. Also very high rates of vitamin D insufficiency found in women of child-bearing age living in Beijing and Hong Kong were detected [22]. A lower serum 25(OH)D level was measured in Tunisia with lower mean level of veiled compared to nonveiled women [23]. In other African countries, studies showed adequate or even high mean serum 25(OH)D levels [24, 25].

Although Oceania has a very sunny climate, studies from Australia, New Zealand, and pacific islands detected low mean 25(OH)D levels (below 20 ng/mL) and large seasonal variation was observed in volunteers in Vercargill, Dunedin, and New Zealand [26]. In USA, Ginde and team in 2009 conducted one of the largest representative samples available which is the National Health and Nutrition Examination Survey (NHANES). It showed that mean serum 25(OH)D levels were 19.9 ng/mL: 12.1 ng/mL in men and 19.8 ng/mL in women [27].

The current study seeks to determine vitamin D serum levels among healthy human volunteers living in Jordan by measuring serum levels of 25(OH)D and to examine the effect of nationality and gender on vitamin D status. In addition, we aimed to find an association between 25(OH)D levels with lipid profile results by determining 25(OH)D serum levels among hyperlipidemia Iraqi out-patients in Jordan.

## 2. Materials and Methods

### 2.1. Study Population

This study was conducted in April 2014 in Jordan. Three hundred and ninety subjects have been enrolled in this study: 195 young (18–30 years) apparently healthy Jordanian and Iraqi subjects and 195 (>40 years) hyperlipidemic Iraqi subjects. Of the 390 subjects, 26.5% were Jordanian and 73.5% were Iraqi. As for the gender, males represent 50.25% and females were 49.75%. In addition, females were subdivided into two groups, first group (50.8%) wearing hijab (hijab is a veil that covers head, arms, and chest) and second group (49.2%) without hijab wearing modernized western style clothes.

The study protocol and the case report form were approved by the research committee (December 2013) at the Faculty of Pharmacy, University of Petra, Amman, Jordan. The case report form (CRF) was used to gather demographics, body mass index (BMI), dress style, vitamin D supplements hypolipidemic medication intake, and type of food intake. Females were categorized according to their dress style, hijab (covering all body parts except the face and hands) and western dress style. Subjects taking vitamin D supplements or under hypolipidemic medications were excluded.

### 2.2. Blood Sampling and Laboratory Analysis

Case report forms were filled by the subjects before blood sampling. Blood samples were collected using 10 mL syringes, transferred into 10 mL plane test tube, stood for clotting for 5–10 minutes, and then centrifuged (Hermle Z320, Hermle labor technique, Germany) at 5000 rpm for 5 minutes. Serum was collected in a separate tube and stored at −70°C until analysis.

### 2.3. Measurement of Vitamin D

Quantitative colorimetric immunoenzymatic determination of 25(OH) vitamin D concentrations in human plasma level was developed by using vitamin D ELISA kit (Diametra, Milano, Italy). The kit is a competitive solid phase enzyme-linked immunosorbent assay (ELISA). Samples were analyzed according to the manufacturer guidelines.

### 2.4. Vitamin D Classifications

Vitamin D levels were classified into 3 major groups according to the classification of the Institute of Medicine (IOM) [28, 29] as follows:

1. sufficient (>30 ng/mL);
2. insufficient (20–30 ng/mL);
3. deficient (<20 ng/mL).

### 2.5. Data Analysis

Data from 390 subjects were expressed as mean ± SD and statistically analyzed using SPSS v. 19.0 for Windows Software Package (SPSS Inc., Chicago, IL, USA). Analytical procedures include correlation analysis to analyze demographic and clinical factors associated with vitamin D levels after logarithmic transformation. The probability value of *P* < 0.050 was considered significant.

## 3. Results

### 3.1. Vitamin D Levels of University of Petra Students

Table 1 summarizes the age and the body mass index (BMI) of the first group subjects (195 young subjects (18–30 years)); 103 subjects were Jordanian (51.46% males, 29.13% females wearing hijab, and 19.42% females wearing western style clothing) and 92 were Iraqi (51.10% males, 28.26% covered females, and 20.65% uncovered females). The impact of nationality, gender, and dress style on vitamin D levels is illustrated in Table 2, in which 32.6% of Iraqis having sufficient, 51.0% having insufficient, and 16.4% having deficient levels of vitamin D compared to 7.7%, 49.5%, and 42.7% for Jordanian, respectively.

**Figure 1:**
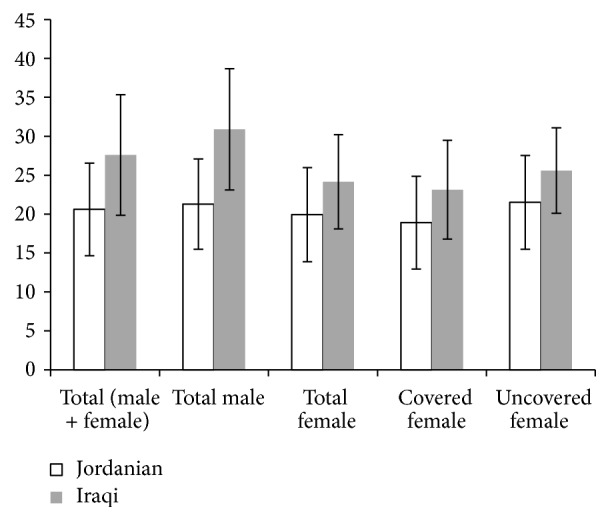
Mean value of vitamin D levels in ng/mL in University of Petra students according to their nationality.

Herein, Figure 1 showed that there was a significant (*P* < 0.050) difference in vitamin D mean level between the total Jordanians (20.60 ± 5.94 ng/mL) and the total Iraqis (27.59 ± 7.74 ng/mL). Also, Iraqi females showed significant higher vitamin D levels than Jordanian females (24.16 ± 6.06 ng/mL versus 19.92 ± 6.06 ng/mL, resp.; *P* < 0.050). Young Iraqi covered females showed higher (23.12 ± 6.34 ng/mL) levels than those of young Jordanian covered female (18.91 ± 5.97 ng/mL) (*P* < 0.050). Same pattern (*P* < 0.050) was observed between young Jordanian western style wearing females and young Iraqi uncovered females (21.52 ± 6.02 ng/mL versus 25.58 ± 5.49 ng/mL, resp.) (Figure 1). Significant differences (*P* < 0.050) in vitamin D levels were detected between the total male students (25.82 ± 8.33 ng/mL and the total female students (21.95 ± 6.39 ng/mL. In contrast, vitamin D levels between Iraqi and Jordanian male students showed no significant difference.

### 3.2. Vitamin D Levels for Healthy and Hyperlipidemic Iraqi Individuals at Ibn Alhaytham Hospital

195 (>40 years) Iraqi subjects; 78 healthy subjects; and 117 hyperlipidemic patients were enrolled in the second part of the study. The age of 78 healthy subjects ranged from 40 to 72 years with the average of 54.3 ± 10.3 years and body mass index (BMI) ranged from 18 to 37 kg/m^2^, with the of average of 27.8 ± 4.2 kg/m^2^ (Table 1). Besides, the age of 117 hyperlipidemic patients ranged from 40 to 75 years, with the average of 54.89 ± 9.39 years, and body mass index (BMI) ranged from 17 to 37 kg/m^2^, with the average of 27.79 ± 4.94 kg/m^2^ (Table 1). Further classification is also found in Table 1.

The data in Table 3 illustrated that 46.15% of healthy subjects had sufficient vitamin D levels (36 subjects; 14 males, 9 hijab wearing females, and 13 western style wearing females), 41.03% subjects had insufficient vitamin D levels (32 subjects; 11 males, 8 covered females, and 13 uncovered females), and 10 healthy old subjects (12.82%) had deficient vitamin D level (2 males, 6 covered females, and 2 uncovered females). Also, about half of the hyperlipidemic patients (51.28%) had vitamin D level between 21 and 29 ng/mL; while 29.06% were with vitamin D deficiency. The rest 19.66% were with sufficient vitamin D level (Table 3).

In the current study, healthy individuals and hyperlipidemic patients were classified according to BMI into 3 major groups: normal (18.5–24.9 kg/m^2^), overweight (25−29.9 kg/m^2^), and obese (>30 kg/m^2^). Of the healthy individuals (*n* = 78), 16.8% were normal, 42.4% were overweight, and 40.8% were obese. Of the 32 obese, and according to vitamin D levels, it was found that 15.3% of them had sufficient, 19.2% had insufficient, and 6.4% had deficient vitamin D levels, while in hyperlipidemic individuals (*n* = 117) 8.5% were normal, 25.6% were overweight, and 41.8% were obese. In 49 of the obese hyperlipidemic patients, it was found that 3.4% of them had sufficient vitamin D level, 18.8% had insufficient vitamin D level, and 19.6% had deficient vitamin D level.

Furthermore, Table 4 showed the differences in vitamin D levels between the healthy and hyperlipidemic Iraqi subjects. A significant difference (*P* < 0.050) was detected between Iraqi healthy males (32.18 ± 10.21 ng/mL) and females (28.55 ± 8.96 ng/mL).

Herein, comparison between healthy and hyperlipidemic patients based on BMI was done and vitamin D mean level for the normal BMI healthy individuals was 30.32 ± 9.42 ng/mL, while that of hyperlipidemic patients was 26.78 ± 9.05 ng/mL. Also, vitamin D level for the overweight BMI healthy individuals was 31.82 ± 11.43 ng/mL, and that of hyperlipidemic patients was 25.47 ± 7.81 ng/mL (Table 4). A significant difference (*P* < 0.050) between obese subjects of the two groups was detected; vitamin D mean level of the obese BMI category healthy individuals was 27.51 ± 6.79 ng/mL, whereas that of hyperlipidemic patients was 20.96 ± 6.42 ng/mL.

The study finding revealed that vitamin D level was decreased with age in both healthy and hyperlipidemic patients as shown in Table 4; vitamin D mean levels for healthy individuals were 37.41 ± 9.09 ng/mL in the age 40–50 years, 31.05 ± 4.54 ng/mL in the age 51–60 years, and 21.51 ± 3.41 ng/mL in the ages >60 years. Similarly, in hyperlipidemic individuals of the age 40–50 years, vitamin D mean level was 25.87 ± 9.13 ng/mL, while that of the age >60 years was 18.69 ± 5.18 ng/mL. It is interesting that for all age groups vitamin D mean levels were significantly higher in healthy compared to hyperlipidemic individuals.

## 4. Discussion

The finding of this study revealed that vitamin D levels are affected by many factors such as nationality, gender, sex, BMI, physical activity, and lifestyle and this was reported previously in Middle East rejoin [30–35].

Also, physical activity and lifestyle are important factors in determining vitamin D level. Herein, Figure 1 showed that there was a significant (*P* < 0.050) difference in vitamin D mean level between the total Jordanian and total Iraqi. Also, Iraqi females showed significant higher vitamin D levels than Jordanian females (*P* < 0.050). Young Iraqi covered females showed higher levels of vitamin D than those of young Jordanian covered female (*P* < 0.050). Same pattern (*P* < 0.050) was observed between young Jordanian western style wearing females and young Iraqi uncovered females (Figure 1). The reason behind those results might be the different lifestyles and physical activity [13, 36]. Clothes are a main blocker to sun exposure and therefore 25(OH)D synthesis and status; in this study, we found that females with western style wearing have higher levels of 25(OH)D than those wearing hijab. Sun exposure to uncovered face and hands as in hijab dressed females is not enough for vitamin D synthesis. In addition, the differences in 25(OH)D levels among covered or uncovered Iraqis or Jordanian females might be related to the texture of clothing; some clothes such as wool, silk, and polyester are blocking UV radiation greater than cotton and linin. In addition, colors may affect the absorption of UV radiation (black is higher absorber than white) [37, 38]. Also, food consumption like dairy product is of the main factors that affect 25(OH)D levels. Moreover, this variation may be related to the latitude of the city or country the participants come from; however, such factor is not addressed in the study since all participants were living in Jordan at the time of the study. Healthy lifestyle is usually associated with sufficient vitamin D serum levels, while the low physical activity is associated with low 25(OH)D levels [39, 40]. In contrast, vitamin D levels between Iraqi and Jordanian male students showed no significant difference, and this may result from the similarity in lifestyles, physical activity, and diet between both groups.

Regardless of the nationality, significant differences (*P* < 0.050) in vitamin D levels were detected between total male students and total female students, which might be explained by the outdoor activity of males, that is, the sun light exposure duration which is more than that of females, and also may be due to the use of sun block by females [41] and breast-feeding in nursing mothers [42]. Excess adipose tissue of females compared with males has been suggested as a causal factor to lower 25(OH)D concentrations in females [43].

The age and BMI of the 195 (>40 years) Iraqi healthy and hyperlipidemic subjects from Ibn Alhaytham Hospital enrolled in the second part of the study are shown in Table 1 and their vitamin D levels are presented in Table 3. Furthermore, Table 4 showed the differences in vitamin D levels between the healthy and hyperlipidemic Iraqi subjects. A significant difference (*P* < 0.050) was detected between Iraqi healthy males and females.

As vitamin D is a fat soluble hormone, thus adipose tissue might be a site of sequestration of vitamin D, storing and subsequently lowering circulating levels of 25(OH)D [1, 3]. And because normal and overweight subjects have adipose tissue less than that of obese subjects, they might show elevation in the availability of vitamin D [44, 45] and this is in accordance with the results revealed in this study.

The inverse relationship between circulating levels of 25(OH)D with risk biomarkers and high lipid profile was detected by many studies [46, 47]. Herein, comparison between healthy and hyperlipidemic patients based on BMI was done and vitamin D mean level for the normal BMI healthy individuals and hyperlipidemic patients was also evaluated. Vitamin D level for the overweight BMI healthy individuals and that for hyperlipidemic patients was presented in Table 4. These findings are consistent with Brock et al., who reported that body mass index (BMI) >30 kg/m^2^ is one of the major factors that affect vitamin D levels [48].

In addition, age is essential factor that affects vitamin D levels like obesity, gender, and diseases. Generally, elder people are susceptible to vitamin D deficiency due to many risk factors, not only due to reduced skin production of vitamin D with age but also due to decreased sunlight exposure, decreased dietary intake, reduced skin thickness, impaired intestinal absorption, and diminished hydroxylation in the liver and kidney [49, 50].

The study finding revealed that vitamin D level was decreased with age in both healthy and hyperlipidemic patients as shown in Table 4 and it is interesting that for all age groups vitamin D mean levels were significantly higher in healthy compared to hyperlipidemic individuals.

The elevation of plasma vitamin D levels in the body is dependent on the vitamin D hydroxylase, vitamin D binding protein (group-specific component; GC), and the inactivation by cytochromes P450 CYP24 (or 25(OH)D-24-hydroxylase) and CYP3A4. Consequently, single nucleotide polymorphism (SNP) markers in the genes, namely, CYP2R1 and GC, might be a reason for different vitamin D levels in healthy Caucasians [51, 52]. And this could be one of the reasons that lead to variation in vitamin levels among different populations. Further research is required in order to clarify the genetic architecture underlying 25(OH)D plasma concentrations among Jordanians and Iraqis.

However, the incidence of vitamin D insufficiency and deficiency in Arab people is multifactorial involving gender, age, obesity, clothing, cultural behaviors, skin pigmentation, vitamin D, calcium supplements, sun exposure, and polymorphism of vitamin D receptors [28, 53–56].

## 5. Conclusion

The current study showed that there was a significant difference in vitamin D concentrations between the total Jordanian and total Iraqi students and there were insignificant differences between Jordanian male and Iraqi students. Vitamin D levels also were significantly different among total Jordanian female and total Iraqi female students and the same results were detected for Jordanian hijab wearing female compared to Iraqi hijab wearing female students. A significant difference was found between Jordanian western styles wearing female students compared to Iraqi uncovered female students.

In addition, overweight and obese BMI categories showed a significant difference between healthy individuals and hyperlipidemic patients though; normal BMI category showed no significant difference between the two groups. In this study, vitamin D levels for healthy individuals were higher than vitamin D levels for hyperlipidemic patients in the three age categories. Hyperlipidemia is associated with decreased vitamin D concentrations through an unknown mechanism. Further studies are needed to replicate these data in larger populations and to elucidate the mechanisms involved in this association. Also, it is necessary to take supplements especially for those who have low plasma 25(OH)D levels related to SNPs markers of inactivating enzymes and/or vitamin D binding protein

## Figures and Tables

**Table 1:** Demographics data (age and body mass index) of all participants (*n* = 390).

Parameter	University of Petra students (*N* = 195)	Old Iraqi healthy individuals at Ibn Alhaytham Hospital (*N* = 78)	Old Iraqi hyperlipidemic patients at Ibn Alhaytham Hospital (*N* = 117)
	Mean ± SD	Mean ± SD	Mean ± SD
Total			
Age (years)	21.51 ± 2.81	54.31 ± 10.30	54.89 ± 9.39
BMI (kg/(m^2^))	24.01 ± 4.12	27.80 ± 4.21	27.79 ± 4.94
Males			
Age (years)	21.82 ± 3.04	54.92 ± 10.22	53.84 ± 8.96
BMI (kg/(m^2^))	25.43 ± 4.43	28.53 ± 4.21	27.94 ± 4.93
Total females			
Age (years)	21.19 ± 2.53	54.01 ± 10.51	57.28 ± 10.02
BMI (kg/(m^2^))	22.51 ± 3.17	27.39 ± 4.23	27.74 ± 5.01
Uncovered females			
Age (years)	21.03 ± 1.81	54.64 ± 10.20	55.56 ± 9.70
BMI (kg/(m^2^))	21.79 ± 2.79	27.31 ± 4.00	26.56 ± 4.99
Covered females			
Age (years)	21.27 ± 2.94	56.45 ± 10.91	58.65 ± 10.25
BMI (kg/(m^2^))	23.00 ± 3.37	28.64 ± 4.76	28.20 ± 5.03

**Table 2:** Distributions of participants from the University of Petra according to gender and nationality.

	Sufficient > 30		Insufficient 20–30		Deficient < 20	
	Number	%	Number	%	Number	%
Jordanian						
Males	4	7.55%	27	50.94%	22	41.51%
Total females	4	8.00%	24	48.00%	22	44.00%
Females covered	1	3.33%	14	46.67%	15	50.00%
Females uncovered	3	15.00%	10	50.00%	7	35.00%
Iraqi						
Males	25	53.19%	18	38.30%	4	8.51%
Total females	5	11.11%	29	64.44%	11	24.44%
Females covered	1	3.85%	17	65.38%	8	30.77%
Females uncovered	4	21.05%	12	63.16%	3	15.79%

**Table 3:** Distributions of participants from Ibn Alhaytham Hospital according to gender, health status, and vitamin D levels.

	Sufficient > 30		Insufficient 21–29		Deficient < 20	
	Number	%	Number	%	Number	%
Healthy						
Males	14	51.85%	11	40.74%	2	7.41%
Total females	22	43.14%	21	41.18%	8	15.69%
Females covered	9	39.13%	8	34.78%	6	26.09%
Females uncovered	13	46.43%	13	46.43%	2	7.14%
Patient						
Males	16	19.75%	42	51.85%	23	28.40%
Total females	7	19.44%	18	50.00%	11	30.56%
Females covered	2	10.00%	12	60.00%	6	30.00%
Females uncovered	5	31.25%	6	37.50%	5	31.25%

**Table 4:** Mean value of vitamin D levels in ng/mL for >40 years of age in healthy and hyperlipidemic Iraqi volunteers.

Category	Healthy	Patient	*P* value
	Vitamin D (ng/mL)	Vitamin D (ng/mL)	
Total	29.78 ± 9.49 (*N* = 78)	23.88 ± 7.93 (*N* = 117)	<0.0001^*∗*^
Total males	32.18 ± 10.21 (*N* = 27)	24.03 ± 8.01 (*N* = 81)	<0.0001^*∗*^
Total females	28.55 ± 8.96 (*N* = 51)	23.53 ± 7.84 (*N* = 36)	0.0224^*∗*^
Normal BMI weight	30.32 ± 9.42 (*N* = 13)	26.78 ± 9.05 (*N* = 29)	0.2208
Overweight (*N* = 33)	31.82 ± 110.43 (*N* = 33)	25.47 ± 7.81 (*N* = 38)	0.0077^*∗*^
Obese	27.51 ± 6.79 (*N* = 32)	20.96 ± 6.42 (*N* = 49)	0.0002^*∗*^
40–50 years	37.41 ± 9.09 (*N* = 29)	25.87 ± 9.13 (*N* = 43)	<0.0001^*∗*^
51–60 years	31.05 ± 4.54 (*N* = 24)	26.34 ± 6.34 (*N* = 39)	0.0027^*∗*^
Over 60 years	21.53 ± 3.41 (*N* = 25)	18.96 ± 5.18 (*N* = 35)	0.0125^*∗*^

^*∗*^Significant (*P* value < 0.050).
